# Similarities and differences in the response and molecular characteristics of peripheral sensory neurons associated with pain and itch: DRG neurons respond differently to pain or itch stimuli

**DOI:** 10.3724/abbs.2024202

**Published:** 2025-02-14

**Authors:** Li Liu, Jiemin Yin, Youqiang Meng, Congrui Ye, Junhui Chen, Sa Wang, Wen Yin, Po Gao, Yingfu Jiao, Weifeng Yu, Yinghui Fan

**Affiliations:** 1 Department of Anesthesiology Renji Hospital School of Medicine Shanghai Jiao Tong University Shanghai 200127 China; 2 Key Laboratory of Anesthesiology (Shanghai Jiao Tong University) Ministry of Education Shanghai 200127 China; 3 Department of Neurosurgery Chongming Hospital Affiliated to Shanghai University of Medicine and Health Sciences Shanghai 200127 China

**Keywords:** *in vivo* two-photon calcium imaging, dorsal root ganglion (DRG), pain and itch stimuli, single-cell RNA sequencing

## Abstract

Dorsal root ganglion (DRG) neurons are responsible for the primary detection and transmission of peripheral noxious stimuli, mainly pain and itch. However, as two distinct noxious sensations, how DRG neurons respond differently to and code pain and itch is still an attractive topic. Here, we investigate the response and activation spectrum of DRG neurons under peripheral pain and itch stimuli using *in vivo* two-photon calcium imaging and find differences in the response intensity to pain and itch between multisensory neurons (both pain and itch) and single-sensory neurons (either pain or itch). In addition, single-cell RNA sequencing (scRNA-seq) is used to reveal the heterogeneity of distinct subpopulations on the basis of their expressions of pain- or itch-related marker genes and to determine the similarities and differences in their transcriptomic changes under chronic pain and itch. Our results show that primary sensory neurons with different sensory patterns respond differently to the same nociceptive stimuli. Additionally, distinct clusters of neurons exhibit unique transcriptomic changes in the development of chronic pain and itch, which may offer new insights for treating these conditions.

## Introduction

Pain and itch are two distinct but equally distressing sensory experiences that have a profound impact on individuals’ emotions and quality of life [ 1, 2] . As they share a similar conduction pathway in the peripheral nervous system, both pain and itch stimuli activate specific receptors on sensatory neurons within the dorsal root ganglion (DRG), initiating action potentials, and then these signals are transmitted to the spinal cord, conveying sensations of pain and itch [1].

In the past, different models of somatosensory discrimination have been proposed, such as specificity, stimulus intensity, firing patterns of afferent nerves, specific subgroups of neurons for each sensory modality, and the spatial arrangement of stimuli [ 3, 4] . However, to date, the encoding characteristics of pain and itch in peripheral sensory neurons remain unclear. In 2009, Liu *et al*. [5] reported that the removal of MrgprA3 ^+^ neurons from the DRG resulted in a specific reduction in itch behavior, indicating that MrgprA3 ^+^ neurons are itch-specific neurons and suggesting the presence of a peripheral itch-labelled line. However, Sharif *et al*. [6] recently demonstrated that metabotropic Gq-linked stimulation of MrgprA3 ^+^ neurons triggers itch, whereas fast ionotropic stimulation of MrgprA3 ^+^ neurons through ChR2 or native P2X3 evokes pain, implying that MrgprA3 C-afferents display intrinsic multimodality. Furthermore, gradually developing genetic, modern molecular, behavioral studies and single-cell transcriptomics indicated that nociceptors are polymodally responsive to multiple stimuli and illustrated the diversity of sensory types and the cellular complexity underlying somatic sensation [ 5, 6] .

In the present study, we transfected mouse DRGs with a virus to express the Ca ^2+^ indicator GCaPM6s and performed *in vivo* two-photon imaging of DRGs, which revealed that there were distinct neuronal subpopulations based on whether they were activated by pain or itch stimuli and revealed their heterogeneity in calcium signal intensity. In addition, we used high-throughput scRNA-seq to investigate the molecular expression characteristics of distinct neuronal subpopulations and their potential functions. Moreover, we analyzed the transcriptional changes in different types of neurons during chronic pain and itch. This study revealed heterogeneity in sensory neuron responses to pain and itch at both the cellular and transcriptomic levels, suggesting the potential roles of distinct somatosensory neurons in the development of chronic itch and pain.

## Materials and Methods

### Animals

Adult male C57BL/6J mice obtained from the vivarium of Shanghai Jiaotong University School of Medicine (Shanghai, China) were used in all the experiments. The animals weighed 22–26 g and were housed in a temperature-controlled room (22–25°C) illuminated from 07:00 to 19:00. Food and water were available *ad libitum*. The present study was performed in accordance with the Guiding Principles in the Care and Use of Animals and the Animal Management Rule of the Ministry of Public Health, People’s Republic of China (documentation 545, 2001) and approved by the Ethics Committee for Experimental Use of Animals of Shanghai Jiaotong University School of Medicine (approval code: #SYXK-2013-0050).

### Surgical procedures for chronic constriction injury (CCI)

The CCI surgery was performed according to established protocols [7]. All surgeries were carried out under gaseous anesthesia using a mixture of 2% isoflurane and oxygen. Initially, the left thigh area of each mouse was carefully shaved and sterilized with iodine solution. The sciatic nerve was subsequently exposed through a blunt incision in the biceps femoris muscle of the left paw. The muscle and fascia surrounding the sciatic nerve were delicately removed without causing stress to the nerve itself. With the use of 4-0 chromic gut sutures, the left sciatic nerve was gently ligated at four points, each with a 1-mm interval. Finally, the muscle and skin layers were closed via 3-0 silk sutures, and iodine solution was applied topically to the skin.

### AEW (acetone-ether-water) itch modeling

The mice were anesthetized with isoflurane and then secured on the surgical table. The left hind leg of each mouse was shaved and disinfected. Acetone and ether were mixed at a 1:1 ratio and then applied to the skin of the mouse’s left hind leg via a 1 cm × 1 cm piece of gauze or cotton ball for approximately 15 s. This was followed by the application of clean gauze or cotton balls soaked in pure water for 30 s. This procedure was performed twice a day with at least a 6-h interval between applications. After 5 days, the AEW itch model was established. The mice in the AEW dry skin itch model exhibited noticeable leg chewing behavior (itching behavior) and skin inflammation.

### DRG exposure surgery and virus injection

First, the mice were anesthetized with 2% isoflurane and then maintained under 1% isoflurane. A 37°C heating pad was placed under the mice to maintain body temperature. After the surgical site was shaved, the skin was disinfected with iodine solution. A longitudinal incision was made on the dorsal skin at the L3–L6 spinal levels, and the muscles on both sides of the L3–L6 spinal processes were excised. Next, virus injection was performed on the left L4 DRG, and a bone clip of the spinal cord adapter was used to secure the L3 vertebral segment to stabilize the spine. Under a microscope, the muscles and fascia attached to the left transverse process of L4 were carefully removed to minimize bleeding during DRG exposure. The left transverse process of L4 was gradually removed with forceps until the L4 DRG was exposed. Under the microscope, a microinjection needle attached to a stereotaxic apparatus was slowly moved until the glass electrode filled with virus was fully inserted into the DRG, and a noticeable rebound of the depressed DRG tissue was observed. The electrode was then gently raised to create a visible depression at the DRG insertion site, and virus injection was initiated at a rate of 800 nL/10 min. The needle was kept in place for 10 min after injection before removal. The adeno-associated virus (AAV)2/9-CAG-GCaMP6s-WPRE-pA used in this study was provided by Shanghai Taitool Bioscience Technology Co., Ltd. (Shanghai, China).

### *In vivo* calcium imaging of the L4 DRG

The surgical procedure for exposing the left L4 DRG was consistent with the aforementioned protocol. Following exposure of the L4 DRG, bone clips at the head and tail ends of the spinal cord adapter were used to secure the L3 and L5 vertebral segments, respectively, completely immobilizing the L4 DRG to ensure stable visualization during *in vivo* calcium signal collection in mice. The spinal cord adapter carrying the mouse was subsequently transferred to the stage of the two-photon microscope (Olympus, Tokyo, Japan). Nociceptive mechanical pinch stimuli were applied to the left hindpaw of each mouse via the toothed forceps as previously described [8]. Mechanical itch is assessed by using a wide range of light mechanical stimuli, such as wool fibres, cotton wisps, brushes, and von Frey filaments, which activate low-threshold mechanoreceptors (LTMRs) [ 9– 11] . In this study, we used a soft wool brush to gently brush the surface of the mouse’s hindfoot, ensuring that the bristles remain as straight as possible during the process to maintain gentle pressure. Changes in the fluorescence intensity of DRG neurons under various peripheral pain and itch stimuli were recorded using 920 nm laser excitation. There was a 5-min interval between two mechanical stimuli to prevent sensitization. To avoid mutual interference from chemical stimuli, there was an interval of at least 40 min between two chemical stimuli, and each mouse received a maximum of two chemical stimuli.

### *In vivo* calcium imaging data analysis

Image acquisition was performed using Olympus FV31S-SW software, with laser imaging parameters that allow for repeated imaging of the same cells without causing damage to the cells or surrounding tissue. The recorded image sequences were exported in TIFF format and motion-corrected using the TurboReg plugin in ImageJ software. The dynamic calcium signal measurements of the ROI were subsequently conducted using the Time Series Analyzer V3 plugin: ΔF/F _0_  = (Ft−F _0_)/F _0_  × 100%, where F _0_ represents the baseline fluorescence signal averaged over the first 60 s of Ft. The activation criterion for DRG neurons was defined as ΔF/F _0_  ≥ 20%.

### Tissue dissociation and cell isolation

L4‒5 DRGs were collected from adult mice (8 weeks old) and placed in cold complete saline (CSS) solution containing specific concentrations of various salts and buffering agents. The DRGs were subsequently subjected to enzymatic digestion via incubation in an enzyme mixture composed of Liberase TM (Roche, Basel, Switzerland) and EDTA at 37°C for specified durations. The enzymatic digestion process involved two stages with different enzyme compositions. Following digestion, the enzymatic activity was halted by adding a solution containing DRG media supplemented with bovine serum albumin (BSA; Sigma, St Louis, USA) and a trypsin inhibitor (Sigma Aldrich). The digested DRG tissue was then gently triturated and centrifuged to obtain a cell suspension. This suspension was passed through a 70-μm cell strainer to remove debris and larger cell aggregates. The resulting cell suspension was centrifuged at 300 *g* for 5 min again to remove excess supernatant, and the pelleted cells were treated with red blood cell lysis buffer to eliminate red blood cells. After wash with phosphate-buffered saline (PBS) containing BSA, the cell pellets were resuspended in PBS with BSA and filtered through a 35-μm cell strainer to obtain a single-cell suspension. Finally, the dissociated single cells were stained with acridine orange/propridium iodide (AO/PI) for viability assessment using a Countstar Fluorescence Cell Analyzer (Inno-Alliance Biotech, San Diego, USA).

### Single-cell RNA sequencing

The scRNA-Seq libraries were prepared using the 10× Genomics Chromium Controller Instrument and Chromium Single Cell 3′ V3.1 Reagent Kits (10× Genomics, Pleasanton, USA). Initially, the cells were concentrated to approximately 1000 cells/μL and introduced into each channel to form single-cell gel beads-in-emulsion (GEMs). Following the reverse transcription (RT) step, the GEMs were disrupted, and barcoded cDNA was isolated and amplified. Subsequently, the amplified barcoded cDNA underwent fragmentation, A-tailing, adaptor ligation, and index PCR amplification. The final libraries were quantified via the Qubit high-sensitivity DNA assay (Thermo Fisher Scientific, Waltham, USA), and their size distribution was assessed with a high-sensitivity DNA chip on a Bioanalyzer 2200 (Agilent, Santa Clara, USA). Finally, all the libraries were sequenced on an Illumina sequencer (Illumina, San Diego, USA) with a 150-bp paired-end run.

### Thresholding method to determine the fraction of positive cells

Single-cell RNA-seq data exhibit considerable noise and are prone to a significant number of false negatives, which can complicate the interpretation of traditional summary statistics such as the mean or median expression levels. To summarize the expression of genes within groups of cells (for example, “cell types”), we employed a thresholding approach [6] aimed at assessing the proportion of cells expressing specific genes. First, we defined the “maximum” expression level for each gene as the average of the top three genes with the highest expression. We subsequently set a threshold for gene expression at 5% of this “maximum” level. Finally, we determined the fraction of cells expressing a particular gene above this threshold ( Supplementary Figure S1).

### Single-cell RNA statistical analysis

The scRNA-seq data analysis was conducted by NovelBio Co., Ltd. (Shanghai, China) utilizing the NovelBrain Cloud Analysis Platform ( www.novelbrain.com). Initially, we employed fastp with default parameters to filter out adaptor sequences and eliminate low-quality reads, thereby obtaining clean data. The feature-barcode matrices were subsequently generated by aligning reads to the mouse genome (mm10, Ensembl100) using CellRanger v5.0.1. Downsampling analysis was applied across samples on the basis of the mapped barcoded reads per cell of each sample, leading to the creation of an aggregated matrix. After quality filtering, cells exhibiting more than 500 expressed genes and a mitochondrial UMI rate less than 20% were retained, whereas mitochondrial genes were excluded from the expression table.

After filtering, we applied the Seurat v4 pipeline [12] for downstream analysis. The data were normalized and scaled via the SCTransform function in which mitochondrial genes were regressed out. Next, the SelectIntegrationFeatures, PrepSCTIntegration, and FindIntegrationAnchor functions were used to identify highly varied cell-to-cell features and “anchors” for the integration of individual datasets. Finally, we obtained an unbatched dataset that integrated all the distinct datasets with the IntegrateData function, and this “batch-corrected” dataset allowed us to analyze all the cells together without potential batch effects. PCA was conducted on the integrated dataset using the RunPCA function on the basis of the scaled and transformed data of identified variable genes. UMAP was performed for visualization using the RunUMAP function.

We clustered cells using the FindNeighbors and FindClusters functions, with the resolution set to 1 in the first round of clustering to identify major cell types, and neurons were reclustered using the abovementioned pipeline with a 0.4 resolution value. After clustering the cells, we identified mostly differentially expressed genes (DEGs) of a certain cluster compared with the remaining clusters with the FindConservedMarkers function with only.pos = TRUE and logfc.threshold > 0.1 as the thresholds to identify markers of a cluster, and the cluster was annotated manually on the basis of the markers. To detect DEGs across the CCI and AEW, we employed FindMarkers with *P*  < 0.05, Log _2_(fold-change) > 0.25 and minimum percentage (min.pct) > 0.1 as the thresholds. The DEGs were enriched for overrepresented biological processes and pathways via clusterProfilers [13].

### Differential gene expression analysis

To detect DEGs across samples, we employed the FindMarkers function using the Wilcoxon rank sum test algorithm, applying the following criteria: 1) *P*  < 0.05; 2) Log _2_(fold-change) > 0.25; and 3) minimum percentage (min.pct) > 0.1.

### Enrichment analysis

Following differential expression analysis, DEGs were selected for Gene Ontology (GO) and Kyoto Encyclopedia of Genes and Genomes (KEGG) enrichment analysis using the R package clusterProfile v4.14.3. Visualization of enriched terms and pathways was performed using the web-based tool ( www.sangerbox.com/tool.html). These analyses highlighted key biological processes and pathways likely contributing to the distinct functional roles.

## Results

### Sensitivity spectrum of DRG neurons in response to pain and itch stimuli

**Figure 1:**
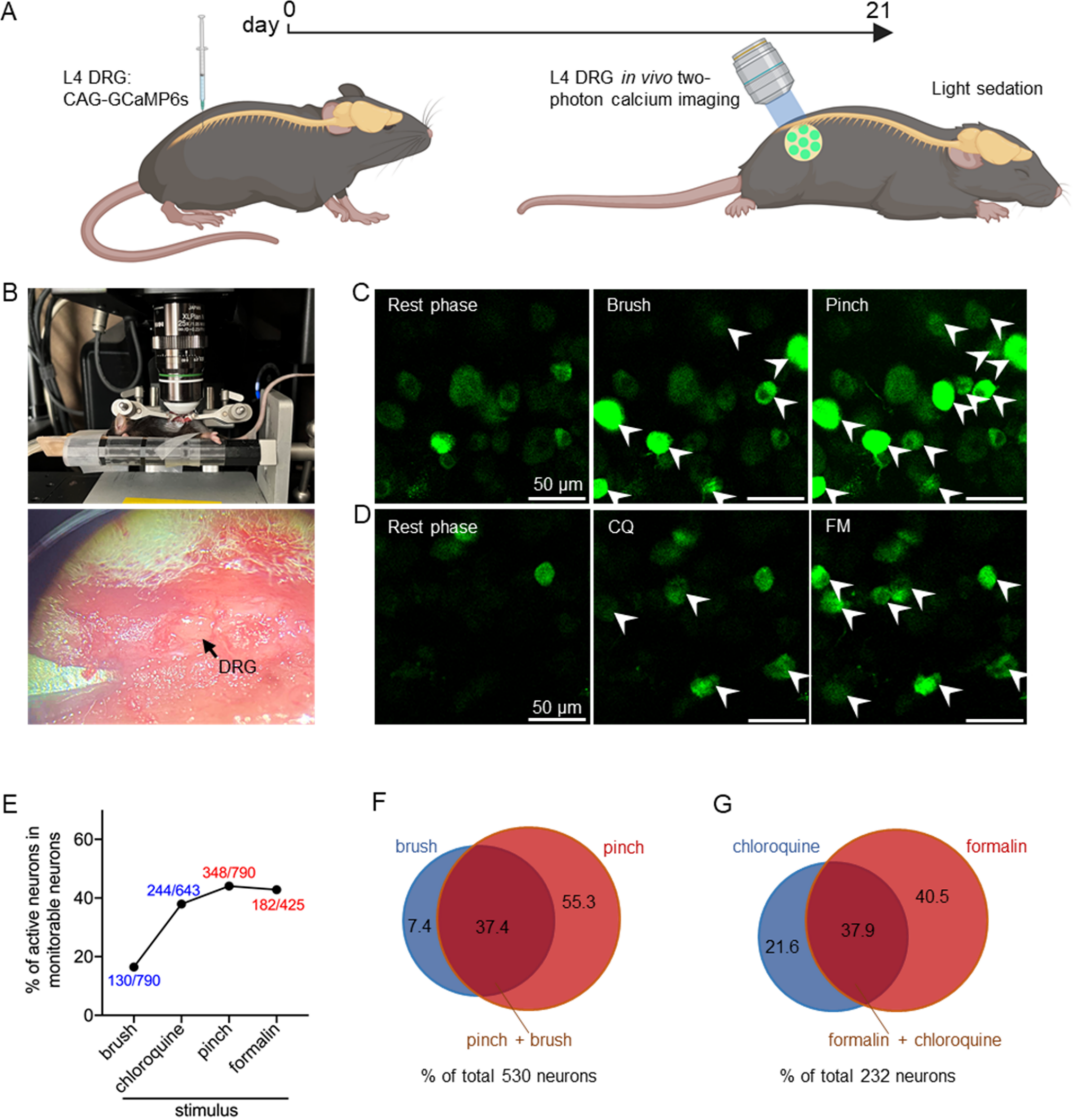
Figure 1 Sensitivity spectrum of DRG neurons in response to pain and itch stimuli (A) Schedule for virus injection of CAG-GCaMP6s in the DRG and in vivo two-photon imaging experiments. (B) Schematic diagram of in vivo two-photon imaging and DRG tissue under a microscope. (C) Representative in vivo two-photon images of DRG neurons obtained before (left panel) and during hind limb 10-s brush (middle panel) and 10-s pinch (right panel) stimulation. Scale bar: 50 μm. (D) Representative in vivo two-photon images of DRG neurons obtained before (left panel), during hind limb chloroquine (100 μg/ 10 μL) injection (middle panel) and after 10 μL of 2% formalin injection (right panel). (E) The percentages of DRG neurons activated by brushes, chloroquine, pinches and formalin. (F) Euler diagrams showing overlaps in neurons responsive to brush and pinch stimuli. The numbers indicate the percentage of neurons responding to at least one of the two types of stimuli, as indicated. (G) Euler diagrams showing overlap in neurons responsive to chloroquine injection and formalin injection.

The L4 DRG receives a large amount of nerve innervation in the hind paw; therefore, we injected an adeno-associated virus expressing the Ca ^2+^ indicator CGaMP6s into L4 DRGs and conducted *in vivo* two-photon calcium imaging of L4 DRGs ( Figure 1A,B). We applied mechanical pain (pinch) and itch (brush) stimuli ( Figure 1C), as well as chemical pain (formalin, subcutaneous injection) and itch (chloroquine, intradermal injection) stimuli ( Figure 1D), respectively, to the plantar surface of the hind paws of the mice to monitor the proportion of activated DRG neurons. Among all detectable DRG neurons, including neurons expressing GFP fluorescence constitutively and neurons activated by peripheral stimulation, the proportions of neurons activated by brush, pinch, chloroquine, and formalin stimuli were 16.5%, 44.1%, 37.9%, and 42.8%, respectively ( Figure 1E).

Next, we analyzed the overlap of DRG neurons activated by pinch and brush stimuli within the same fields of view. Among the 530 neurons activated by pinch or brush stimuli, 37.4% of the neurons responded to both pinch and brush stimuli (198/530), 55.3% of the neurons responded exclusively to pinch stimuli (293/530), and only 7.4% of the neurons were activated exclusively by brush stimuli (39/530) ( Figure 1F). In addition, we observed the activity of neurons in the same fields of view for 11 min after chloroquine or formalin administration. To exclude the influence of the injection procedure on neuronal calcium signals, we overlooked the data collected within 1 min after drug injection. Among the 232 neurons activated by chloroquine or formalin, 37.9% of the neurons responded to both chloroquine and formalin (88/232), 21.6% of the neurons responded exclusively to chloroquine (50/232), and 40.5% of the neurons were activated only by formalin (94/232) ( Figure 1G).

### Differential *in vivo* calcium responses of DRG neurons to pain and itch stimuli

**Figure 2:**
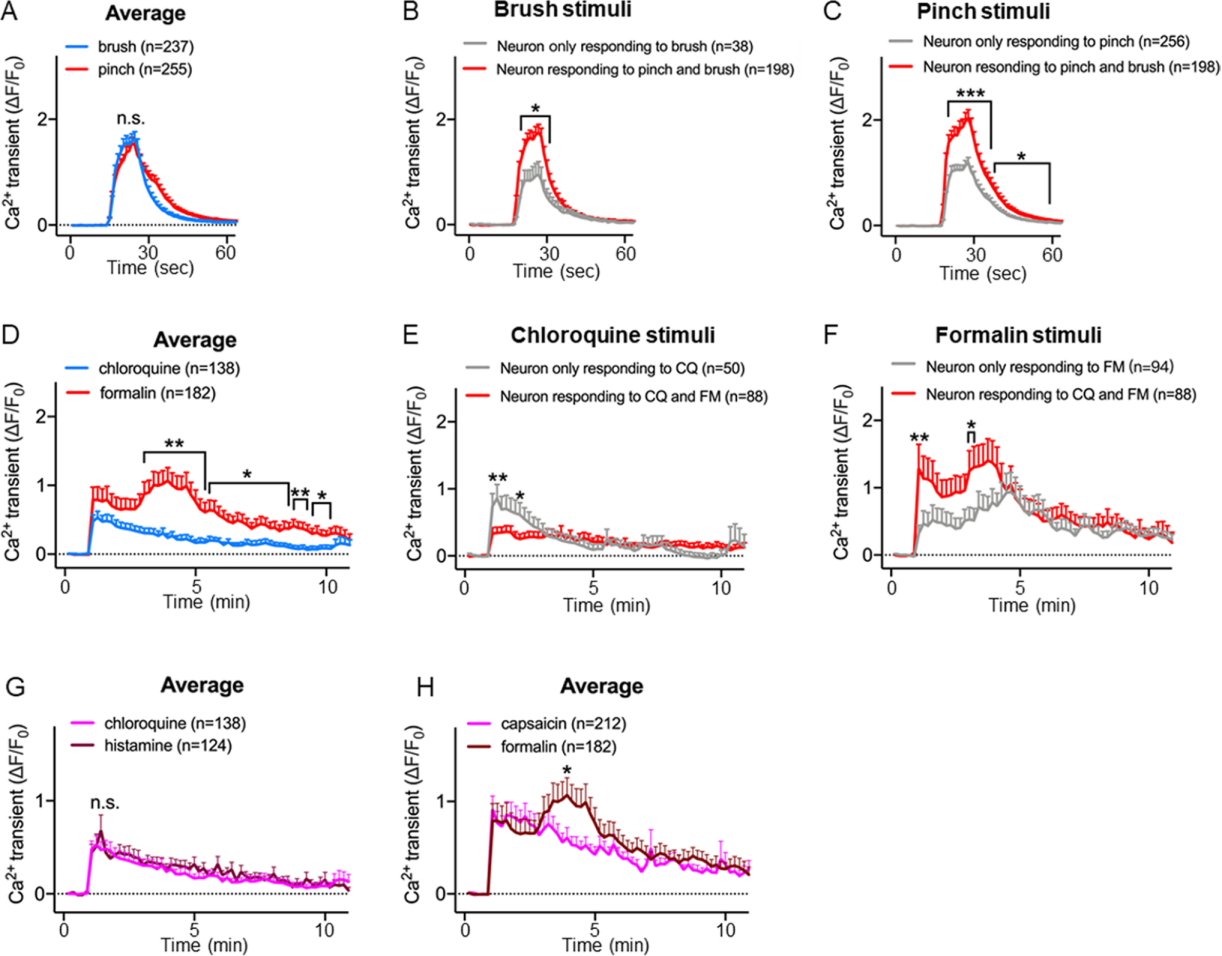
Figure 2 Differential *in vivo* calcium responses of DRG neurons to pain and itch stimuli (A) Population mean ΔF/ F0 of activated sensory neurons following brushing and pinching. (B) Population mean ΔF/ F0 induced following exposure of neurons that respond to both pinch and brush stimuli and neurons that respond only to brush stimuli. (C) Population mean ΔF/ F0 induced following pinch stimuli of neurons that respond to both pinch and brush stimuli and neurons that respond only to pinch stimuli. (D) Population mean ΔF/ F0 of activated sensory neurons following chloroquine and formalin treatment. (E) Population mean ΔF/ F0 induced following chloroquine injection of neurons that respond to both chloroquine and formalin and neurons that respond only to chloroquine. (F) Population mean ΔF/ F0 induced following formalin injection of neurons that respond to both chloroquine and formalin and neurons that respond only to formalin. (G) Population mean ΔF/ F0 of activated neurons following chloroquine or histamine injection. (H) Population mean ΔF/ F0 of activated neurons following capsaicin or formalin injection. CQ, chloroquine; FM, formalin. * P < 0.05, **P < 0.01, ***P < 0.001; unpaired t test. Data are presented as the mean ± SEM.

To explore the response of DRG neurons to pain and itch stimuli of the same nature in greater depth, we analyzed the population of calcium transients of neurons in response to mechanical pain (pinch) and itch (brush) stimuli. We found no significant difference in the amplitude of Ca ^2+^ transients between neurons activated by pinching and those activated by brushing ( Figure 2A). However, the neurons activated by both stimuli presented significantly greater amplitudes of Ca ^2+^ transients to either pinch or brush than did the neurons activated by only one type of stimulus ( Figure 2B,C).

Next, formalin and chloroquine were injected into the hind paw as chemical pain and itch stimuli, respectively. The calcium signals of neurons activated by formalin were significantly stronger than those of neurons activated by chloroquine ( Figure 2D). Additionally, the duration of activation of neurons induced by formalin was longer ( Figure 2D). Furthermore, compared with neurons activated only by formalin, neurons that responded to both stimuli presented significantly greater Ca ^2+^ responses to formalin ( Figure 2F). However, neurons that responded exclusively to chloroquine presented significantly greater Ca ^2+^ responses to chloroquine than did neurons that responded to both stimuli ( Figure 2E).

Next, we investigated the calcium responses of DRG neurons to various chemical itch stimuli, namely, chloroquine and histamine. The calcium transients induced by these two itch inducers did not differ ( Figure 2G). However, by comparing pain stimuli, we found that the calcium transients induced by formalin were significantly greater at approximately 4 min post injection than those induced by capsaicin ( Figure 2H).

### Cellular constitution of the mouse DRG

**Figure 3:**
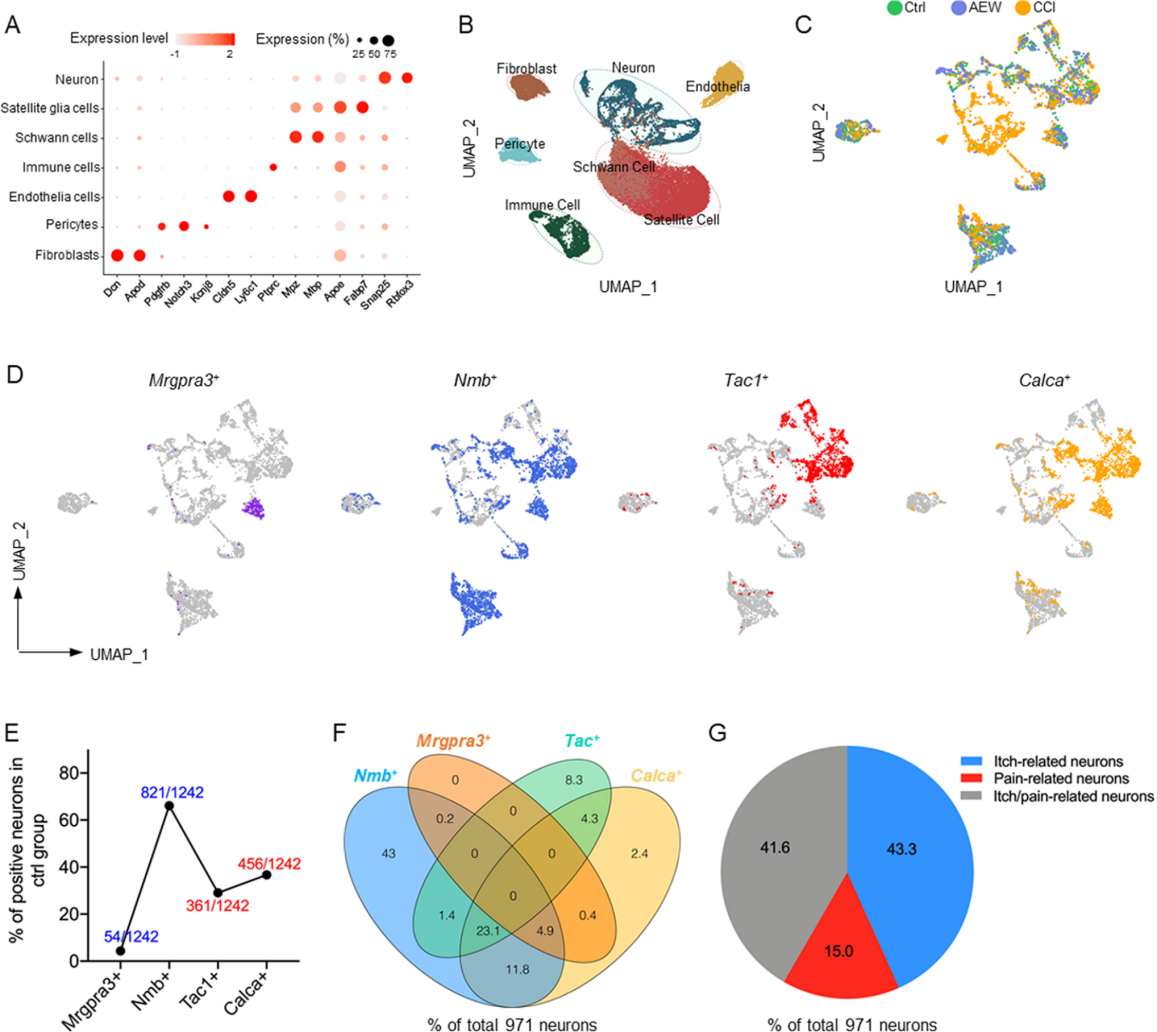
Figure 3 Proportions of pain-, itch- and itch/pain-related neurons revealed by scRNA-seq (A) Bubble plot of the marker genes of 7 DRG cell types. The color represents the average expression level of the marker genes, and the size represents the percentage of cells expressing the marker gene in this cluster. (B) UMAP plot of DRG cells. Each dot represents an individual cell; each cell type is marked by a unique color. (C) UMAP plot showing neurons in the control (green), AEW (purple) and CCI (yellow) groups. Ctrl, control. (D) UMAP plots of control group neurons stained for Mrgpra3 +,Nmb +,Tac1 + and Calca + neurons. (E) The proportions of Mrgpra3 +,Nmb +,Tac1 + and Calca + neurons in control group neurons. (F) Venn diagram showing the overlapping proportions of Nmb +,Mrgpra3 +,Tac1 + and Calca + neurons in a total of 971 neurons. (G) Pie chart showing the proportions of itch-related neurons (blue), pain-related neurons (red), and itch/pain-related neurons (gray).

We conducted scRNA-seq on the cells dissociated from the left lumbar (L) 4-5 DRGs of naïve (control group), AEW itch and CCI pain mice. After quality control, 34,917 cells were obtained from 6 samples (2 control, 2 AEW, and 2 CCI samples). On the basis of gene expression patterns, we mapped all the cells into 7 cell types with distinct markers ( Figure 3A) and classified them by uniform manifold approximation and projection (UMAP) ( Figure 3B). These cells were neurons ( *Rbfox3* ^+^), satellite glial cells ( *Fabp7* ^+^), fibroblasts ( *Dcn* ^+^), pericytes ( *Notch3* ^+^), immune cells ( *Ptprc* ^+^), schwann cells ( *Mpz* ^+^), and endothelial cells ( *Cldn5* ^+^) ( Figure 3B). This classification is consistent with previous reports [14].

### scRNA-seq reveals the proportions of pain- and itch-related neurons

A total of 5089 neurons from the 3 groups were extracted and visualized in UMAP via the Seurat package ( Figure 3C). *Nmb* and *Mrgpra3* are itch-related marker genes, whereas *Tac1* and *Calca* are pain-related marker genes [15]. We employed a threshold-based “fraction of positive cells” measurement method proposed by Usoskin *et al*. [6], which labels *Mrgpra3* ^+^, *Nmb* ^+^, *Tac1* ^+^ and *Calca* ^+^ neurons in control group neurons ( Figure 3D and Supplementary Figure S1), and calculated their proportions in the control group neurons (1242 neurons) as 4.3%, 66.1%, 29.1%, and 36.7%, respectively ( Figure 3E). We calculated the number of all four types of neurons, totaling 971 neurons. The Venn diagram revealed a significant degree of overlap among these neurons ( Figure 3F). To better characterize the neurons expressing markers of pain or itch, we categorized these neurons into three classes: itch-related neurons ( *Mrgpra3* ^+^ or *Nmb* ^+^ neurons), accounting for 43.3% (420/971); pain-related neurons ( *Tac*1 ^+^ or *Calca* ^+^ neurons), accounting for 15% (146/971); and itch/pain-related neurons expressing *Nmb* or *Mrgpra3* along with *Tac1* or *Calca*, accounting for 41.6% (405/971) ( Figure 3G).

### Transcriptomic characteristics of pain-, itch- and itch/pain-related neurons

**Figure 4:**
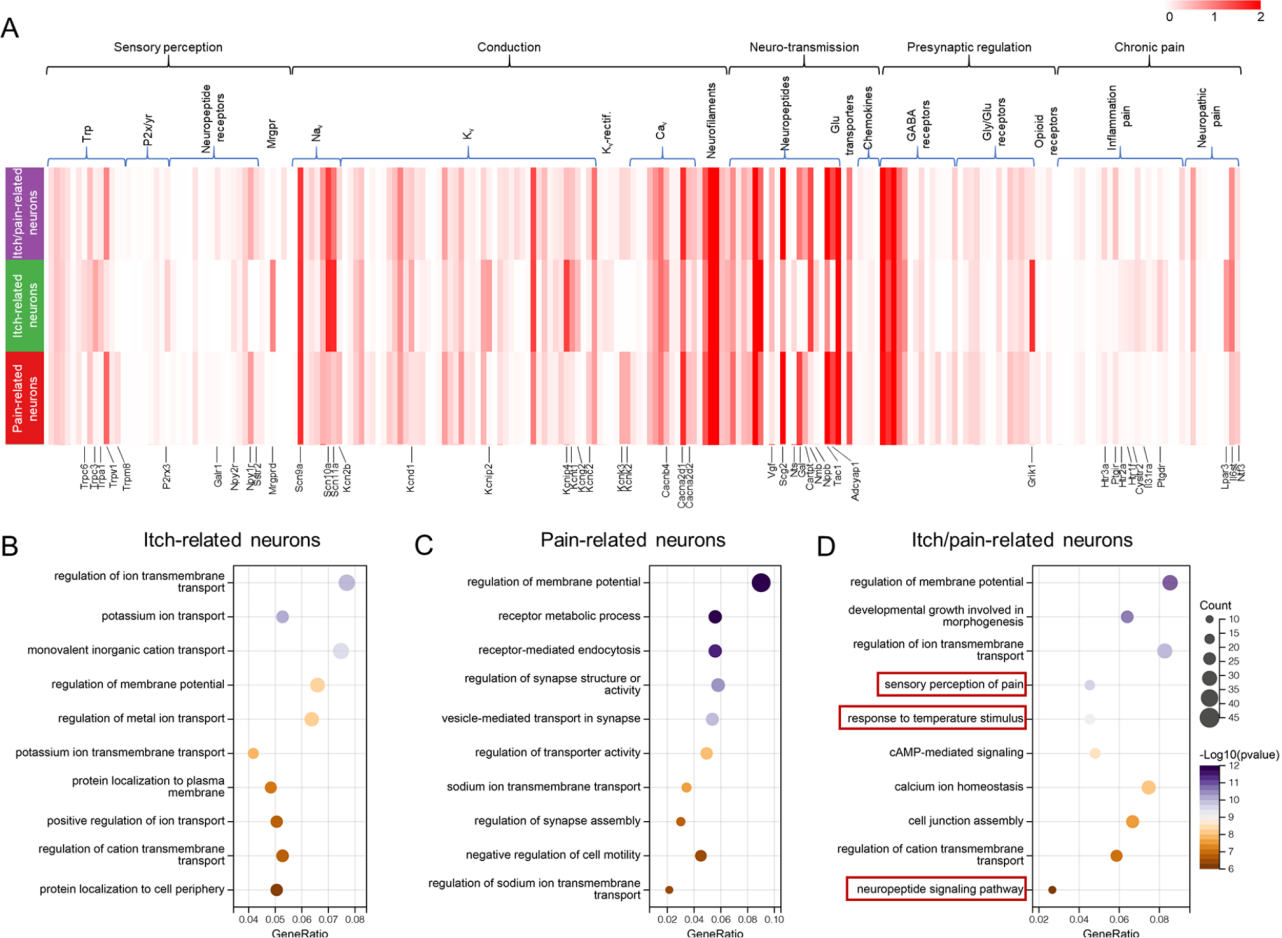
Figure 4 scRNA-seq revealed the transcriptomic characteristics of itch-, pain-, and itch/pain-related neurons (A) Heatmap of the expressions of the various operational components in pain, itch, and itch/pain neurons. The color represents the average expression level of genes. Some DEGs among the three types of neurons are labelled by name in the heatmap. Supplementary Table S1 presents the data presented in this panel. Trp, transient receptor potential cation channel; P2x/yr, purinergic receptor P2X or P2Y; KV-rectif., open rectifier potassium channel (Kcnk); Glu, glutamate; Gly, glycine. (B–D) Bubble plots showing the results of the GO analysis of the biological process terms enriched with the highly expressed DEGs of itch-, pain-, itch/pain-related neurons.

Peripheral stimuli activate receptors on sensory neurons, causing depolarization and action potential conduction mediated by voltage-gated calcium channels (Cav channels), sodium channels (Nav channels), and potassium channels (Kv channels). This leads to the release of signaling molecules such as neuropeptides into the spinal cord. Additionally, local excitation, inhibitory circuits, and presynaptic inhibition of the spinal cord influence the transmission of sensory signals. Analysis of genes reported in the literature to play key roles in sensory transduction revealed the operational component characteristics of these three types of neurons in terms of peripheral stimulus receptors, depolarization, action potential propagation, neurotransmitter release, etc. ( Figure 4A and Supplementary Table S1).

The patterns of highly expressed *P2rx3* and *Mrgprd* in itch-related neurons suggest that these neurons are nonpeptidergic neurons [ 6, 16] . Moreover, these neurons highly express *Trpc3*, *Trpa1*, sodium ion channel genes ( *Scn10a* and *Scn11a*), potassium ion channel genes ( *Kcnd1*, *Kcnip4*, *Kcnt1*, and *Kcng2*), the glutamine receptor gene *Grik1*, and a series of genes associated with itch transmission, such as *Nppb*, *Htr2a*, *Htr1f*, *Cysltr2*, *P2rx3*, *Il31ra* and *Lpar3* [ 6, 17, 18] . The operational component expression patterns of the other two types of neurons are similar. Pain-related neurons and itch/pain-related neurons are peptidergic neurons, defined by the expression of the marker gene *Tac1* [19]. In addition, these two types of neurons contain high levels of calcium ion channel genes ( *Cacna2d1* and *Cacna2d2*), *Trpv1*, *Gal*, and *Adcyap1*. However, compared with itch/pain-related neurons, pain-related neurons presented greater expression of *Trpm8*, potassium ion channel genes ( *Kcn2b*, *Kcn3*, and *Kcn2*), and *Ntf3* and lower expression of *Galr1* and sodium ion channel genes ( *Scn10a* and *Scn11a*).

We next annotated the DEGs associated with biological processes [ *P*  < 0.05, Log _2_(fold-change) > 0.25] from the GO database to obtain the transcriptomic characteristics of these three neuronal subpopulations. Overexpressed genes of itch-related neurons recapitulated membrane potential- and ion channel-related biological processes ( Figure 4B), such as “regulation of ion transmembrane transport,” “potassium ion transport,” “regulation of membrane potential” and “regulation of cation transmembrane transport”. DEGs highly expressed in pain-related neurons were enriched in synapse- and membrane potential-related biological processes, such as “regulation of membrane potential”, “regulation of synapse structure or activity”, “vesicle-mediated transport in synapses” and “regulation of sodium ion transmembrane transport” ( Figure 4C). DEGs with high expression in itch/pain-related neurons were prominently enriched in “regulation of membrane potential”, “regulation of ion transmembrane transport”, “sensory perception of pain”, “response to temperature stimulus”, “calcium ion homeostasis”, “cell junction assembly”, “regulation of cation transmembrane transport”, and “neuropeptide signaling pathway” ( Figure 4D).

### Contribution of itch-, pain-, and itch/pain-related neurons in the DRG to chronic pain and itch

**Figure 5:**
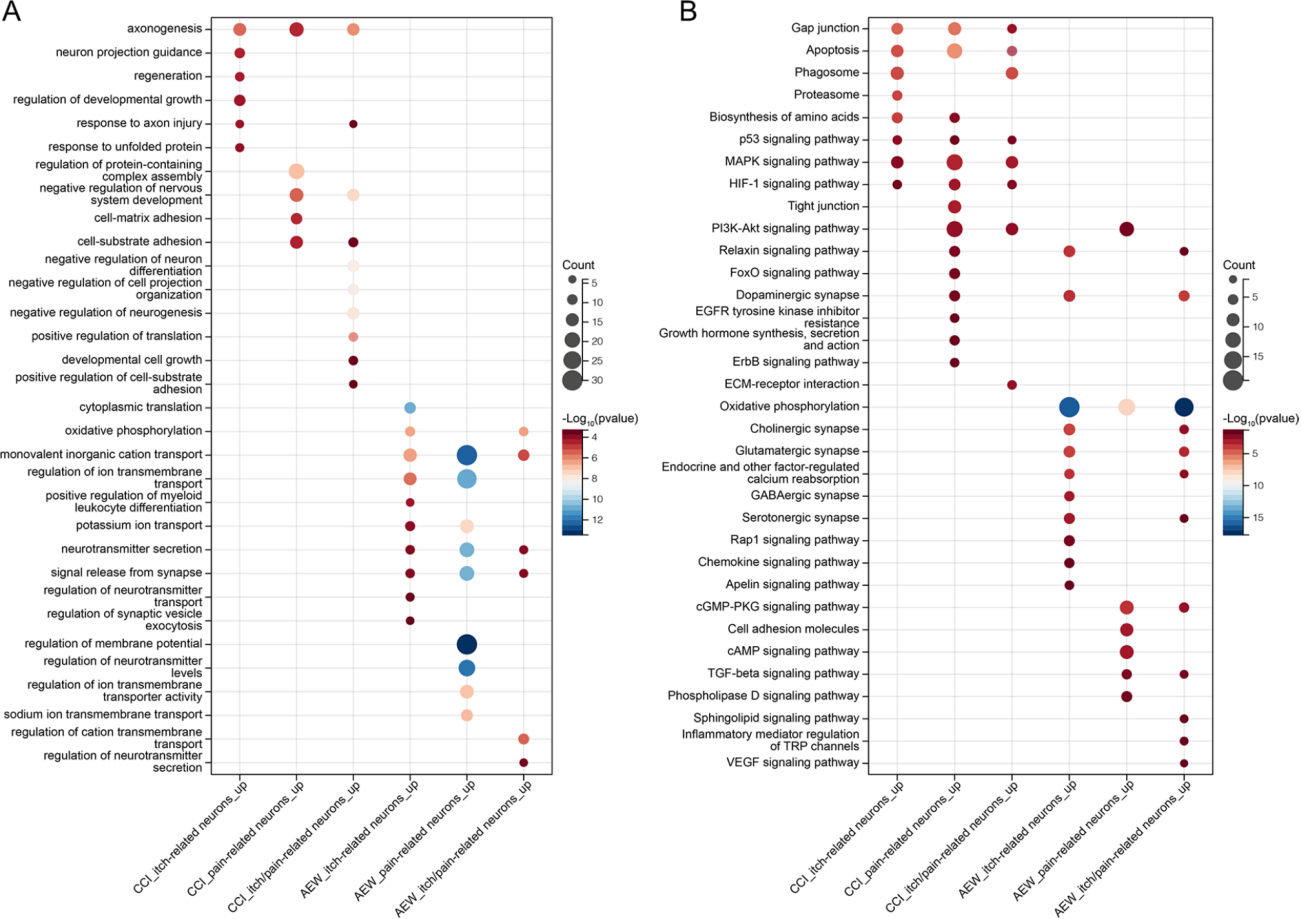
Figure 5 Contribution of DRG neurons to chronic pain or chronic itch (A) The biological process terms of the DEGs highly expressed in the three types of neurons under CCI and AEW conditions. (B) KEGG pathways enriched with DEGs highly expressed in three types of neurons under CCI and AEW conditions.

To explore the potential functional differences among the three neuronal subpopulations under AEW itch and CCI pain conditions, we conducted GO enrichment analysis of their highly expressed genes under the two states [ *P*  < 0.05, Log _2_(fold-change) > 0.25] ( Figure 5A). Under CCI pain conditions, the biological process enrichment of the highly expressed DEGs of these three types of neurons involved neuroregeneration-associated biological processes such as “axonogenesis”. In addition, adhesion-related biological processes such as “cell-substrate adhesion” were enriched in pain-related neurons and itch/pain-related neurons. Additionally, under the AEW itch condition, ion transport-, neurotransmitter-, and signal transmission-related biological processes were enriched in three types of neurons: “monovalent inorganic cation transport”, “neurotransmitter secretion”, and “signal release from synapses”.

Subsequently, KEGG analysis of the DEGs was undertaken ( Figure 5B), revealing that “gap junction”, “apoptosis”, “p53 signaling pathway”, “MAPK signaling pathway”, and “HIF-1 signaling pathway” were preferentially activated across the three types of neurons during CCI pain. Moreover, the “oxidative phosphorylation” pathway was activated across 3 types of neurons in the context of AEW itch.

## Discussion

Itch and pain are distinct sensations of discomfort that can arise from overlapping receptive fields in the skin, prompting investigations into how pruritus and nociception are encoded and differentiated. An increasing number of studies have indicated that peripheral sensory neurons are multimodal [ 6, 20] . For example, MrgprA3 ^+^ neurons, previously suggested as a specific pathway for itch, respond to itch stimuli such as chloroquine (CQ) or chemogenetic activation but evoke pain responses when optogenetically activated to induce rapid ion flux [21]. In this study, we examined the activation of DRG neurons in response to pain or itch stimuli, including their proportion and intensity using *in vivo* two-photon imaging. Additionally, we analyzed the transcriptomic profiles of distinct neuronal subpopulations and their transcriptomic changes following CCI or AEW via scRNA-seq.

In this study, we employed viral transduction of postnatal mouse DRG tissue to express GCaMP6s instead of relying on transgenic mouse lines expressing GCaMP during embryonic development. This approach avoids potential calcium buffering during embryogenesis. Compared with GCaMP6f and GCaMP6m, GCaMP6s is more sensitive, enabling improved monitoring of neuronal calcium activity [ 22, 23] .

Our findings revealed considerable overlap among neurons activated by pain and itch stimuli of the same nature (mechanical brush and pinch, chemical chloroquine and formalin), underscoring the multimodal capacity of peripheral primary neurons. Similarly, previous research by Wang *et al*. [20] demonstrated that a significant portion of DRG neurons respond to multiple types of stimuli, including mechanical and varying degrees of thermal stimuli.

A notable discovery from our study is the distinct calcium transient patterns observed in neuronal populations classified on the basis of their responses to pain or itch stimuli. Compared with neurons exclusively responsive to pain, multisensory neurons respond to both pain and itch stimuli and exhibit pronounced calcium transients under pain conditions. In neurons, the activation of cell surface receptors triggers calcium ion influx through ion channels or induces calcium release from the endoplasmic reticulum, thereby promoting neuronal excitation [24]. This calcium transient extent mediates sensory signal transmission [25]. Hence, multisensory neurons may play a pivotal role in pain transmission. Nevertheless, neurons exclusively responsive to chloroquine presented significantly greater calcium transient amplitudes in response to chloroquine than in response to both chloroquine and formalin. This may be because single-sensory neurons to chloroquine express higher levels of *P2rx3*. Research has shown that P2X3Rs play crucial roles in the itch process induced by chloroquine through MrgprA3 ^+^ neurons [26]. In addition, voltage-dependent Ca ^2+^ channels in neurons are directly involved in the regulation of calcium inflow [ 27– 29] . Therefore, high expression of *Cacnb4* might contribute to itch transmission in neurons that exclusively respond to chloroquine. The distinct calcium response profiles of DRG neurons offer new insights into the classification of peripheral neuronal subtypes.

Furthermore, formalin induced greater calcium transients than did capsaicin approximately 4 min after injection. However, neurons that respond to different chemical itch substances (histamines and chloroquine) exhibit calcium responses that are not significantly different. These findings suggest that DRG neurons may display varying calcium transients in response to diverse pain stimuli, potentially influenced by drug concentration, which could modulate the intensity of calcium responses.

Under naïve conditions, the proportions of neurons identified by scRNA-seq as itch-, pain-, itch/pain-related subpopulations were significantly different from those of neurons responding exclusively to itch stimuli, pain stimuli, or both, as revealed by two-photon calcium imaging. These differences may arise from variations in the transcriptional and translational regulation of genes [30].

scRNA-seq analysis revealed that itch-related neurons expressed marker genes ( *P2rx3* and *Mrgprd*) specific to non-peptidergic neurons, which are known to participate in pruritus [ 17, 31] . Additionally, itch-related neurons highly expressed genes involved in itch transmission, such as *Nppb*, *Htr2a*, *Htr1f*, *Cysltr2*, *P2rx3*, *Il31ra* and *Lpar3*, underscoring the reliability of classifying these neurons as itch-related neurons in this study. Pain- and itch/pain-related neurons are peptidergic and highly express *Tac1*, suggesting their involvement in thermosensitive sensation [ 32– 34] . Consistently, GO analysis predicted that the functional characteristics of itch/pain-related neurons enriched in “response to temperature stimulus”. Furthermore, these neurons are enriched in terms associated with “sensory perception of pain” and “calcium ion homeostasis”, potentially explaining their heightened responsiveness to pain stimuli.

Next, GO enrichment analysis was performed on highly expressed genes in three types of neurons under CCI and AEW conditions. Compared with those in the AEW state, genes highly expressed in the three types of neurons under CCI were enriched in GO terms associated with nerve regeneration, which is consistent with previous reports linking common neuronal regeneration-related transcriptional changes following nerve injuries [ 14, 35– 37] . In addition, GO functions and KEGG pathways related to cell adhesion were significantly enriched in pain-related neurons and itch/pain-related neurons. Indeed, cellular adhesion processes help improve axonal regrowth and neuronal physiology [38]. Under CCI conditions, GO functions enriched in genes highly expressed across the three neuronal subpopulations indicated significant deficits in neurotransmitter secretion, likely reflecting the impact of nerve injury on synaptic vesicles.

In conclusion, we identified distinct neuronal subpopulations responsive to pain or itch stimuli and demonstrated significant differences in calcium signal intensity between multisensory and single-sensory neurons. In addition, we utilized scRNA-seq to analyze the transcriptomic characteristics of these distinct neuronal subpopulations classified on the basis of their expressions of pain- or itch-related markers and examined their transcriptomic changes following CCI or AEW. This study highlights the diversity of sensory types and the cellular complexity underlying somatic sensation and offers new insights for the treatment of chronic pain and itch.

## Supporting information

24470Supplementary_Table_1

24470supplementary_Figure_S1

## Supplementary Data

Supplementary Data is available at *Acta Biochimica et Biophysica Sinica* online.

## References

[1] McGlone F, Reilly D (2010). The cutaneous sensory system. Neurosci BioBehav Rev.

[2] Kini SP (2011). The impact of pruritus on quality of life. Arch Dermatol.

[3] LaMotte RH, Dong X, Ringkamp M (2014). Sensory neurons and circuits mediating itch. Nat Rev Neurosci.

[4] Schmelz M. Itch and pain differences and commonalities.
Handb Exp Pharmacol 2015, 227: 285–301. https://doi.org/10.1007/978-3-662-46450-2_14.

[5] Zhang C, Hu MW, Wang XW, Cui X, Liu J, Huang Q, Cao X (2022). scRNA-sequencing reveals subtype-specific transcriptomic perturbations in DRG neurons of PirtEGFPf mice in neuropathic pain condition. eLife.

[6] Usoskin D, Furlan A, Islam S, Abdo H, Lönnerberg P, Lou D, Hjerling-Leffler J (2015). Unbiased classification of sensory neuron types by large-scale single-cell RNA sequencing. Nat Neurosci.

[7] Liu L, Chen J, Yin W, Gao P, Fan Y, Wen D, Jiao Y (2024). The peripheral Atf3
^+^ neuronal population is responsible for nerve regeneration at the early stage of nerve injury revealed by single-cell RNA sequencing. Acta Biochim Biophys Sin.

[8] Zhang S, Cai B, Li Z, Wang K, Bao L, Li C, Zhang X (2022). Fibroblastic SMOC2 suppresses mechanical nociception by inhibiting coupled activation of primary sensory neurons. J Neurosci.

[9] Sakai K, Akiyama T (2020). New insights into the mechanisms behind mechanical itch. Exp Dermatol.

[10] Andersen HH, Akiyama T, Nattkemper LA, van Laarhoven A, Elberling J, Yosipovitch G, Arendt-Nielsen L (2018). Alloknesis and hyperknesis—mechanisms, assessment methodology, and clinical implications of itch sensitization. Pain.

[11] Bataille-Savattier A, Le Gall‐Ianotto C, Lebonvallet N, Misery L, Talagas M (2023). Do merkel complexes initiate mechanical itch?. Exp Dermatol.

[12] Butler A, Hoffman P, Smibert P, Papalexi E, Satija R (2018). Integrating single-cell transcriptomic data across different conditions, technologies, and species. Nat Biotechnol.

[13] Yu G, Wang LG, Han Y, He QY (2012). clusterProfiler: an R package for comparing biological themes among gene clusters. OMICS-J Integr Biol.

[14] Renthal W, Tochitsky I, Yang L, Cheng YC, Li E, Kawaguchi R, Geschwind DH (2020). Transcriptional reprogramming of distinct peripheral sensory neuron subtypes after axonal injury. Neuron.

[15] Chen ZF (2021). A neuropeptide code for itch. Nat Rev Neurosci.

[16] Dong X, Han S, Zylka MJ, Simon MI, Anderson DJ (2001). A diverse family of GPCRs expressed in specific subsets of nociceptive sensory neurons. Cell.

[17] Han L, Ma C, Liu Q, Weng HJ, Cui Y, Tang Z, Kim Y (2013). A subpopulation of nociceptors specifically linked to itch. Nat Neurosci.

[18] Yin W, Liu L, Zhou Y, Zhang Y, Kong D, Xu S, Tang D (2021). Complete Freund’s adjuvant-induced decrement of pruriceptor-mediated suppression of itch. Acta Biochim Biophys Sin.

[19] O′Connor TM, O′Connell J, O′Brien DI, Goode T, Bredin CP, Shanahan F (2004). The role of substance P in inflammatory disease. J Cell Physiol.

[20] Wang F, Bélanger E, Côté SL, Desrosiers P, Prescott SA, Côté DC, De Koninck Y (2018). Sensory afferents use different coding strategies for heat and cold. Cell Rep.

[21] Sharif B, Ase AR, Ribeiro-da-Silva A, Séguéla P (2020). Differential coding of itch and pain by a subpopulation of primary afferent neurons. Neuron.

[22] Broussard GJ, Liang R, Tian L (2014). Monitoring activity in neural circuits with genetically encoded indicators. Front Mol Neurosci.

[23] Chen TW, Wardill TJ, Sun Y, Pulver SR, Renninger SL, Baohan A, Schreiter ER (2013). Ultrasensitive fluorescent proteins for imaging neuronal activity. Nature.

[24] Wheeler JJ, Davis JM, Mishra SK. A calcium imaging approach to measure functional sensitivity of neurons.
Methods Mol Biol 2022, 2413: 97–106. https://doi.org/10.1007/978-1-0716-1896-7_11.

[25] Sun L, Tong CK, Morgenstern TJ, Zhou H, Yang G, Colecraft HM (2022). Targeted ubiquitination of sensory neuron calcium channels reduces the development of neuropathic pain. Proc Natl Acad Sci USA.

[26] Shiratori-Hayashi M, Hasegawa A, Toyonaga H, Andoh T, Nakahara T, Kido-Nakahara M, Furue M (2019). Role of P2X3 receptors in scratching behavior in mouse models. J Allergy Clin Immunol.

[27] Tsien RW, Tsien RY (1990). Calcium channels, stores, and oscillations. Annu Rev Cell Biol.

[28] Catterall WA (1991). Functional subunit structure of voltage-gated calcium channels. Science.

[29] Ghosh A, Greenberg ME (1995). Calcium signaling in neurons: molecular mechanisms and cellular consequences. Science.

[30] de Klerk E, ‘t Hoen PAC (2015). Alternative mRNA transcription, processing, and translation: insights from RNA sequencing. Trends Genet.

[31] Liu Q, Tang Z, Surdenikova L, Kim S, Patel KN, Kim A, Ru F (2009). Sensory neuron-specific gpcr mrgprs are itch receptors mediating chloroquine-induced pruritus. Cell.

[32] Caterina MJ, Rosen TA, Tominaga M, Brake AJ, Julius D (1999). A capsaicin-receptor homologue with a high threshold for noxious heat. Nature.

[33] McKemy DD, Neuhausser WM, Julius D (2002). Identification of a cold receptor reveals a general role for TRP channels in thermosensation. Nature.

[34] Tominaga M, Caterina MJ, Malmberg AB, Rosen TA, Gilbert H, Skinner K, Raumann BE (1998). The cloned capsaicin receptor integrates multiple pain-producing stimuli. Neuron.

[35] Chandran V, Coppola G, Nawabi H, Omura T, Versano R, Huebner EA, Zhang A (2016). A systems-level analysis of the peripheral nerve intrinsic axonal growth program. Neuron.

[36] Costigan M, Befort K, Karchewski L, Griffin RS, D′Urso D, Allchorne A, Sitarski J (2002). Replicate high-density rat genome oligonucleotide microarrays reveal hundreds of regulated genes in the dorsal root ganglion after peripheral nerve injury. BMC Neurosci.

[37] LaCroix-Fralish ML, Austin JS, Zheng FY, Levitin DJ, Mogil JS (2011). Patterns of pain: meta-analysis of microarray studies of pain. Pain.

[38] Lavdas AA, Papastefanaki F, Thomaidou D, Matsas R (2011). Cell adhesion molecules in gene and cell therapy approaches for nervous system repair. Curr Gene Ther.

