# Supplementary material for: Similarities and differences in the response and molecular characteristics of peripheral sensory neurons associated with pain and itch: DRG neurons respond differently to pain or itch stimuli
Source: Acta Biochim Biophys Sin (Shanghai). 2025 Feb 14;57(6):890–900. doi: 10.3724/abbs.2024202 (PMC12247137; doi:10.3724/abbs.2024202)
Supplement: 24470supplementary_Figure_S1 [file 24470supplementary_Figure_S1.docx]

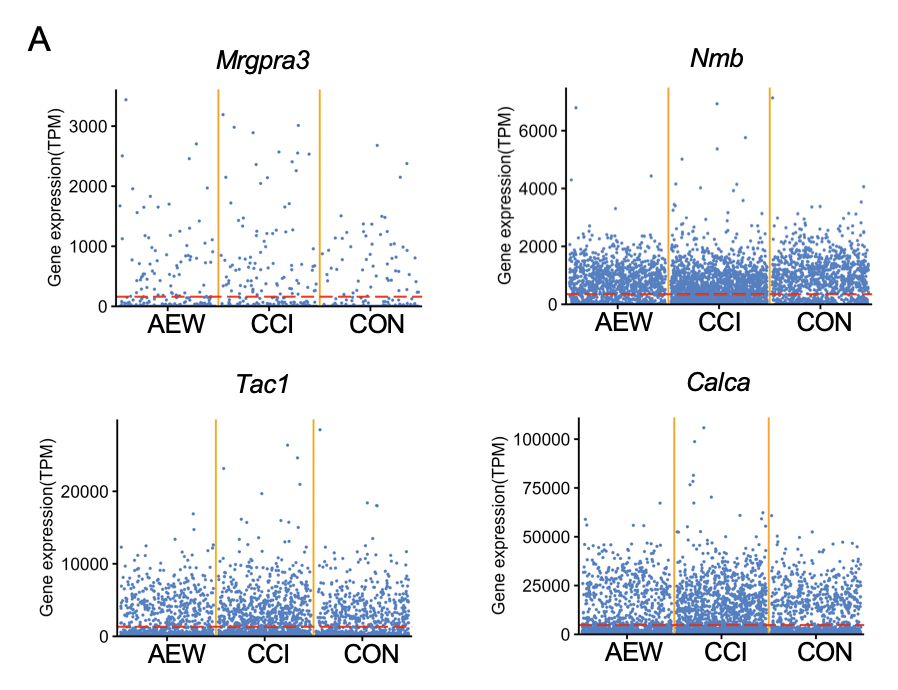


**Supplementary Figure S1. Thresholding method to determine fraction of *Mrgpra3-*, *Nmb*-, *Tac1*-, *Calca*-positive cells**  Thresholding approach was used to summarize the expressions of genes (*Mrgpra3*, *Nmb*, *Tac1*, *Calca*) within neurons. The red dotted line represents the threshold.
